# A Critical Review of Instruments Measuring the Quality of Life of Cancer Patients in Iranian Studies and Their Psychometrics Properties

**DOI:** 10.31557/APJCP.2019.20.2.333

**Published:** 2019

**Authors:** Shirin Hasanvand, Maryam Rassouli, Zahra Mandegari, Naiire Salmani, Farzaneh Moghimkhan

**Affiliations:** 1 *School of Nursing and Midwifery, Social Determinants of Health Research Center, Lorestan University of Medical Sciences, Khorramabad,*; 2 *Cancer Research Center,*; 4 *Medical Library and Information Sciences, Mofid Children Hospital, Shahid Beheshti University of Medical Sciences, Tehran,*; 3 *Meybod Nursing School, Shahid Sadoughi University of Medical Sciences, Yazd, Iran. *

**Keywords:** Quality of life, validity, reliability, cancer, psychometric evaluation

## Abstract

**Objective:**

Over the past three decades, more than 100 instruments have been developed that measure the quality of life. In order to ensure accuracy and authenticity of the measurements, it is essential to utilize the instruments that have proper psychometric properties. Therefore, this critical review study aimed at comparing the instruments that measured the life quality of cancer patients in Iranian studies.

**Methods:**

In this study, Persian articles published in Iranian databases (IranMedex, Irandoc Magiran, SID) from 2006 to 2016 were searched, using the following keywords: nursing, cancer, tools, scale, and quality of life. A total number of 159 articles were obtained, of which 33 articles complied with the inclusion criteria of this study and thereby were reviewed.

**Results:**

Sixty nine percent of the articles explored the life quality of adult females with breast cancer, and the most-commonly used instrument was a quality-of-Life instrument for use in international clinical trials in oncology belonging to the European Organization for Research and Treatment of Cancer. More than half of the studies referred solely to Iranian studies to address psychometric properties. Though, no method was introduced in order to address the validity and reliability of instruments in the articles under consideration.

**Conclusion:**

According to the findings, the studies which explored the validity and reliability of instruments concerning the life quality of cancer patients were scarce. Therefore, the researchers should pay further attention to the validity and reliability of instruments for selection of an appropriate instrument in this area of research. Also, the researchers are encouraged to further study the psychometric properties of relevant instruments so that optimal generalizability and authenticity of their findings can be attained.

## Introduction

Cancer is the third cause of death in Iran (Rouhollahi et al., 2014). According to the GLOBOCAN statistical estimates, the incidence of death due to cancer has been reported over 85,000 in Iranian population, and this figure is predicted to increase even more in the next decade due to the recent lifestyle changes and increasing life expectancy. A recent estimate indicated that the incidence of lung, stomach, breast and prostate cancer has increased from 2001 to 2015 in Isfahan city (Bahrami, 2016). with over 20 million new cancer cases expected annually as early as 2025. it seems likely that cancer will become one of the major health problems in Iran. 

In spite of the efforts that have been devoted to the prevention, screening, and early diagnosis of cancer, the treatment of cancer is mainly invasive and still requires many resources yet. Direct and indirect costs of cancer in Iran have been estimated almost 3 billion dollars a year, which is a considerable number compared with its global rate being 18.1 trillion dollars in 2010 (Ferlay et al., 2015).

Due to the rising number of cancer patients and limitation of the resources in Iran, almost all efforts are being devoted to the promotion of their physical criteria such as increasing the survival of cancer patients or reducing their mortality rates; and other aspects of the patient’s life are being neglected. However, it is well-known that improved quality of life in cancer patients can be achieved not only by meeting their physical needs but also their other needs (Bahrami, 2016).

The quality of life requires an understanding of the individuals’ goals, expectations, interests, and beliefs in their cultural domains (Üstündağ and Zencirci, 2015). The World Health Organization (WHO) also defines the quality of life as the perception of individuals’ living conditions in accordance with their culture, norms, goals, expectations, standards, and interests (Bonomi et al., 2000). 

The quality of life is a multi-dimensional concept that is mostly being evaluated through self-administered instruments which address various facets of the individual’s performance and wellness (Jacobsen et al.,2002). Therefore, it is noteworthy that the concepts vary in accordance with the type of the instrument under consideration (Jacobsen and Jim, 2011). The main aspects of individuals’ quality of life include functional, physical, emotional and social dimensions as well as the disease’s symptoms and therapeutic side effects (Leppert et al., 2015).

In recent years, the life quality monitoring has received considerable attention due to its potential significance in the identification of patients’ challenges and health system’s plans concerning various diseases, particularly the chronic ones (Torkzahrani et al., 2013). In addition, the quality of life is considered as a pivotal factor in order to assess the health system’s outcomes, and is one of the most effective strategies to evaluate the effectiveness of delivered cares to the patients with chronic diseases (Burckhardt and Anderson, 2003). The goals of delivering care to cancer patients not only include increasing their survival but also performing appropriate symptom management and improving their quality of life (Üstündağ and Zencirci, 2015). Subsequently, the life quality is perceived as a significant concept in research as well as clinical practices regarding cancer patients in particular (Tadele, 2015). 

Although more than one hundred questionnaires that evaluate life quality have been developed over last three decades, there is no consensus regarding the appropriateness of the instruments (Jacobsen and Jim, 2011; Burckhardt and Anderson, 2003; Tadele, 2015). However, it is necessary to use the instruments with appropriate psychometric properties in order to ensure the authenticity of measurements (Tamburini, 2001). Given the key role of nurses in delivering care to the patients, it appears important that the instruments with acceptable validity and reliability be used for assessment of the patients’ life quality, identification of high-risk patients, and development of care plans. Accordingly, this critical study aimed at reviewing and comparing instruments that measure the life quality of cancer patients in nursing the literature. 

## Materials and Methods

This was a critical review study in which Persian databases (i.e. IranMedex, Irandoc, Magiran, and SID) were searched from 2006 to 2016 in order to obtain the relevant papers. The keywords which were selected for this purpose included “Nursing”, “Cancer”, “Instrument”, “scale”, and “Quality of life”. The search procedures were performed in accordance with each database’s guideline. 

In an earlier phase of the study, the abstracts were reviewed to evaluate the compatibility of papers and inclusion criteria of this study. The inclusion criteria for the current study included the studies which: (1) had the first author and/or correspondent author as a member of nursing community, (2) were conducted on cancer patients, (3) were written in Persian, and (4) were published between 2006 to 2016. The researches in the form of case report, letter to the editor as well as qualitative and review articles were excluded from this study. Also, the articles without the term “Quality of life” in their studied instruments were excluded as well. 

## Results

A total number of 159 articles were found through literature review of which 33 articles met the inclusion criteria of this study. According to literature review, the studies concerning the quality of life in cancer patients particularly those having breast cancer have become the matter of interest among nursing scholars and researchers from Iran as well as other countries over last decade. In other words, almost %39 of the studies explored the quality of life in female adults who were diagnosed with breast cancer. The results of the literature review are summarized in Table 1.

A number of 20 articles out of total 33 articles used the quality-of-Life instrument for use in international clinical trials in oncology (EORTC QLQ-C30) belonging to the European Organization for Research and Treatment of Cancer (Agha barari et al., 2007; Heravi Karimovi et al., 2006; Poorkiani et al., 2010; Zeighami Mohammadi and Ghaffari, 2009; Hasanvand et al., 2015; Shariati et al., 2011; Zeighami Mohammedi et al., 2008; Momeni and Ghanbari, 2011; Baljani et al., 2011; Tork Zahrani et al., 2012; Ghavam-Nasiri et al., 2012; Sadat Aghahosseini et al., 2012; Davoodi et al., 2012; Sharif et al., 2010; Mardani Hamule and Shahraki Vahed, 2010; Ayatollahi, 2013; Haghighat, 2013; Mikaili, 2014; Taghadosi and Fahimifar, 2014; Yazdani, 2015; Karbaschi et al., 2015). This instrument was nominated as “Aronson’s life quality index” in one of the studied articles (Zeighami Mohammadi and Ghaffari, 2009). Other instruments and their frequency of use in different studies are presented in Table 2.

**Table 1:** The Studies Regarding the Life Quality Assessment

Number	Author(s) (Year)	Title of research study	Type of study	The instrument’s name	The instrument’s history	The instrument’s psychometrics in Iran	The validity and reliability assessment in study
1	Aghabarari et. al. (2007)	Physical, emotional and social dimension of quality of life among breast cancer women under chemotherapy	An analytical-descriptive- cross-sectional study	The quality of life breast cancer version (QOL- BC)	Developed by the City of Hope National Medical Center	No information.	Validity: The instrument was translated into Persian with no explanation on the content validity assessment using the qualitative method as well as the experts’ number and their specialties. Reliability: The split-half method was used and correlation coefficient at 0.76 was obtained.
2	Heravi et. al. (2006)	Study of the effects of group counseling on quality of sexual life of patients with breast cancer under chemotherapy at Imam Khomeini Hospital	A randomized clinical trial study	(EORTC QLQ C-30)	No information	Two studies in Iran confirmed the validity and reliability of the instrument.	No information
3	Tabari et. al. (2007)	Evaluation of the quality of life in newly recognized cancer patients	A descriptive-analytical study	The Beck’s standard questionnaire	No information	No information	References to previously published articles were provided for the instrument’s validity.
4	Fazel et. al. (2008)	The Effect of Mastectomy on Mood and Quality of Life in Breast Cancer Patients	A descriptive-analytical study	The Ferrans and Powers Quality of Life Index (FPQOLI)	No information	No information	The parallel-form reliability and Cronbach’s alpha.
5	Heydari et. al. (2009)	Correlation of perceived social support from different supportive sources and the size of social network with quality of life in cancer patients	A descriptive correlational study	The Ferrans and Powers Quality of Life Index- cancer version (QLI-CV) questionnaire	No information	No information	No information
6	Pourkiani et. al. (2010)	Does a rehabilitation program improve quality of life in breast cancer patients?	A randomized clinical trial study	The quality-of-Life questionnaire belonging to the European Organization for Research and Treatment of Cancer (EORTC QLQ C-30)	No information	The validity and reliability were confirmed by a Persian study and the instrument was confirmed by several studies belonging to the foreign nations.	No information
7	Zeighami Mohammadi et. al. (2009)	Sexual dysfunction and its correlation with quality of life among women affected with cancer	A descriptive–correlative study	The Aronson's life quality index.	No information	The validity and reliability were confirmed by a Persian study and a study in a foreign nation.	No information
8	Mardani Hamooleh et. al. (2010)	The assessment of relationship between mental health and quality of life in cancer patients.	A cross-sectional descriptive correlational study	The 36-item questionnaire of the life quality (SF-36)	No information	The reliability was confirmed by two Persian studies.	The content validity was assessed using the qualitative method and the reliability was assessed through the test-retest method.
9	Samiai et. al. (2010)	The study of the effects of group counseling on symptom scales of QOL of patients with breast cancer undergoing chemotherapy.	A quasi-experimental study	The quality-of-Life questionnaire belonging to the European Organization for Research and Treatment of Cancer (EORTC QLQ C-30) & The life quality questionnaire in patients with breast cancer (EORTC QLQ - BR23)	The instrument belongs to the European Organization for Research and Treatment of Cancer.	The reliability was confirmed by a Persian study and the relevant values were provided for each instrument.	No information
18	Davoodi et. al. (2012)	Effect of Educating Self-Care Program on Quality of Life in Patients with Gastric Cancer after Gastrectomy in Tabriz Hospitals.	A quasi-experimental study	The quality-of-Life questionnaire belonging to the European Organization for Research and Treatment of Cancer (EORTC QLQ C-30)& the European Organization for Research and Treatment of Cancer Quality of Life Questionnaire-Stomach (EORTC QLQ-STO22)	No information	No information	No information
19	Mikaili (2014)	Evaluation of the effect of chemotherapy on functional scales of quality of life of patient with breast cancer	A quasi-experimental study	(EORTC QLQ (EORTC QLQ - BR23)	The instrument belongs to the European Organization for Research and Treatment of Cancer.	The validity and reliability of the instrument were confirmed by referring to two previously published Persian articles.	No information
20	Shariati et. al. (2013)	The impact of relaxation therapy on functional indexes of the life quality of cancer patient undergoing chemotherapy	A quasi-experimental study	(EORTC QLQ C-30) & (EORTC QLQ - BR23)	The instrument belongs to the European Organization for Research and Treatment of Cancer.	Referred to only one Persian study.	The content validity was achieved.
21	Ayatollahi (2013)	Quality of life in breast cancer patients: Study in the Omid cancer research center– Urmia.	A cross-sectional study	(EORTC QLQ C-30)	The instrument belongs to the European Organization for Research and Treatment of Cancer.	The validity and reliability were confirmed by three Persian studies and the instrument was confirmed in the studies of foreign nations.	No information
22	MousaRezayi et. al. (2013)	A Survey on life quality and its relationship with disease characteristics and demographic variables in cancer patients referring to the Oncology Hospital of Isfahan.	A descriptive study	(SF-36)	No information	The validity and reliability were confirmed in a study and the reliability value was reported at %75. The reliability values of other studies were mentioned as well.	Test-retest and the values of previous articles
23	Bahrami et. al. (2013)	Death Anxiety and its Relationship with quality of life in Women with Cancer	A cross-sectional study	The McGill Quality of Life Questionnaire	No information	The instrument was introduced as a standard questionnaire by referring to only one Persian study. The validity and reliability were confirmed by referring to two different studies conducted on the patients with AIDS and cancer and without any other details.	The instrument is translated with no explanation on the content and construct validity as well as the results. The reliability is assessed through test-retest method and the correlation coefficient at 0.72 is achieved.
24	Haghighat (2013)	The effect of Reflexology on Quality of Life of breast cancer patients during chemotherapy	A randomized clinical trial study	(EORTC QLQ C-30) & (EORTC QLQ - BR23)	The instrument belongs to the European Organization for Research and Treatment of Cancer.	References to two Persian studies were provided with no information about the reliability in both studies.	
25	Sharif et. al. (2009)	The effect of peer-led education on the life quality of mastectomy patients referred to breast cancer-clinics in Shiraz, Iran	A quasi-experimental study study	(EORTC QLQ C-30) & (EORTC QLQ - BR23)	No information	The instrument was used in several studies in foreign nations and references to two Persian papers were provided.	No information
26	Monfared et. al. (2013)	Health-Related Quality of Life and its related factors among women with breast cancer	An analytical descriptive study	(SF-36)	No information	Reference to a Persian study confirming the instrument was provided.	No information

**Table 2:** Frequency of the Instruments Evaluating the Life Quality of Patients with Cancer

Instruments evaluating the life quality of patients with cancer	Frequency
The quality-of-Life questionnaire belonging to the European Organization for Research and Treatment of Cancer (EORTC QLQ C-30)	20
The 36-item questionnaire of the life quality (SF-36)	4
The Functional Assessment of Cancer Therapy – General (FACT-G)	2
Ferrans and Powers Quality of Life Index (FPQOLI)	2
The McGill Quality of Life Questionnaire	1
The life quality questionnaire belonging to the National Medical Center And Beckman Research Institute	1
The Fatigue Symptom Inventory (FSI)	1
The Beck’s standard inventory	1
The Functional Assessment of Cancer Therapy-Colorectal (the FACT-C)	1

**Diagram 1:**
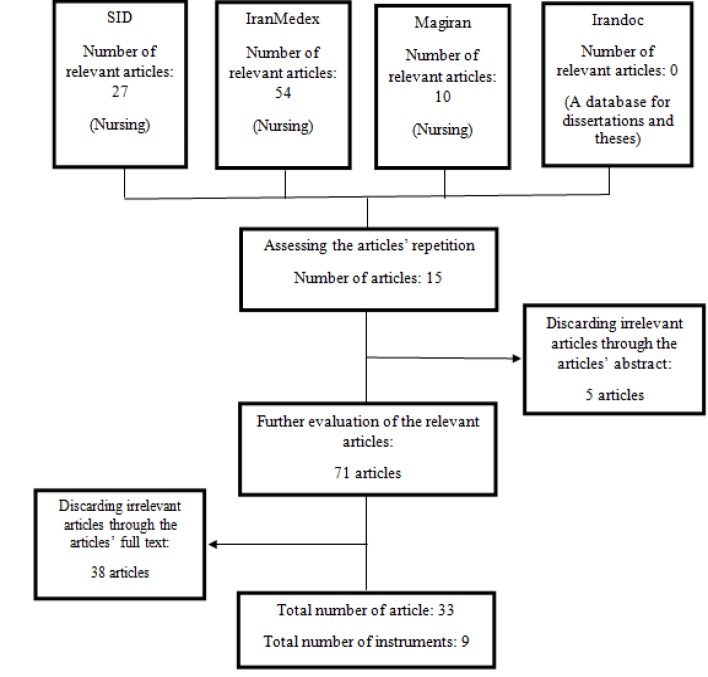
The Process of Paper Selection

In less than half of the studies (%39), the instrument’s history was briefly explained. However, these information were missed in the remaining studies (%61) (Agha barari et al., 2007, Hasanvand et al., 2015, Shariati et al., 2011, Momeni and Ghanbari, 2011, Ghavam-Nasiri et al., 2012, Sharif et al., 2010; Mardani Hamule and Shahraki Vahed, 2010; Ayatollahi, 2013; Haghighat, 2013, Taghadosi and Fahimifar, 2014; Yazdani, 2015, Rad et al., 2016; Heydari et al., 2009). As to the instrument’s history, the researcher(s) merely pointed to the instrument’s author(s) and, in some cases, the year it was designed. However, there was no explanation about the instrument’s design process and its accuracy. In 58 percent of the articles, psychometric properties including validity and reliability were explained by citation of Persian articles. In most studies, references to the articles were provided with no additional information concerning their validity and reliability details. Hence, 32 percent of the studies lacked citation to the references. In 55 percent of the studies, no method was used to determine the validity and reliability of the instruments (Heravi Karimovi et al., 2006; Poorkiani et al., 2010; Zeighami Mohammadi and Ghaffari, 2009; Hasanvand et al., 2015; Shariati et al., 2011; Zeighami Mohammedi et al., 2008; Momeni and Ghanbari, 2011; Sadat Aghahosseini et al., 2012; Davoodi et al., 2012; Sharif et al., 2010; Ayatollahi, 2013; Haghighat, 2013; Taghadosi and Fahimifar, 2014; Yazdani, 2015; Karbaschi et al., 2015; Rezaei et al., 2013; Monfared et al., 2013; Abedi et al., 2014; Tabari et al., 2007). Only 11 studies provided information regarding instrumental validity and reliability. In two studies, the explanations concerning validity and reliability of the instruments were improper. For instance, a study referred to the books and previously published studies for determination of instrumental validity and reliability (Bahrami et al., 2013). Another study pointed to the content validity method for the explanation of reliability (Ayatollahi, 2013). In four studies, the authors stated that the instruments were translated; However, the translation processes were explained unclearly (Agha barari et al., 2007, Heydari et al., 2009, Shahsavari et al., 2015; Fazel et al., 2008). 

Content validity was merely a brief explanation of the usage of qualitative methods for determination of content validity and taking account of the experts’ opinions with no reasoning on their selection, number, and detailed process. The majority of the studies used only a single method for determination of reliability. Only two studies used at least one method for determination of instrumental validity and reliability (Rad et al., 2016; Samiei Siboni et al., 2010). 

## Discussion

In today’s world, the number of studies in medical and social domains that use instruments such as questionnaires, checklists, educational tests, and peer feedbacks are increasing (Cook and Beckman, 2006). In this regard, the validity and reliability of instruments are inevitable concepts that are required to be in an acceptable condition (Drost, 2011; Golafshani, 2003). According to previous studies, most studies had flaws in reporting the validity and reliability properties and provided insufficient information in this regard (DeVon et al., 2007).Quality of life is a multidimensional concept that has no distinct definition due to the fact that it involves a wide range of social, environmental, psychological and physical values (Wells et al., 2011; Theofilou, 2013). More than one thousand instruments have been developed so far in order to evaluate this concept (Ubel et al., 2003).Therefore, this study aimed at reviewing the instruments that evaluate the life quality of cancer patients in Iranian nursing researches in a critical manner. 

The findings revealed that the EORTC QLQ C-30 instrument was the most commonly used tool in the majority of research studies. Some studies used general instruments (e.g. SF-36, HOQOL) and some other used specific instruments in accordance with the type of disease (e.g. breast, colorectal, cervical, and gastric cancers). These are consistent with the findings of a systematic review study by Wheelwright et al., (2013) that explored the quality-of-life instruments in cancer patients with cachexia. According to their study, 33 studies (%49) out of 67 studies used the EORTC QLQ C-30 questionnaire. Fitzsimmons et al., (2009) in their systematic review article studied the life quality instruments utilized in the elderly patients with cancer. The EORTC QLQC-30 questionnaire was used in 12 studies out of total 31 studied articles. 

Another systematic review study by Damm et al., (2013) also reviewed the clinical trial studies that explored the quality of life in patients with lung cancer. Their study revealed that out of 43 studies, 5 studies used a general quality-of-life instrument, 5 studies used a questionnaire that is exclusively developed for cancer patients, and 4 other studies used different instruments which were specifically used in patients with lung cancer. In 29 studies, at least 2 instruments assessing the quality of life were used that each comprised a general and a specific instrument. Also, another finding revealed that the most commonly used instrument was the EORTC QLQ C-30 questionnaire which was in agreement with our study.

Compared with the previously mentioned findings, however, a systematic review study by Cornish et al., (2009) that explored the life quality in patients with dermal melanoma revealed that 20 different instruments were used in 13 studied articles. The most commonly used instruments were SF-36 (5 articles out of 13 studied articles) and EORTC QLQ C-30 (5 articles out of 13 studied articles) questionnaires. Three other articles used an instrument consisting of multiple questionnaires and one study used a questionnaire which was specifically developed for patients with dermal melanoma. It is noteworthy that a general instrument was used in half of the studies articles which was in contrast with our findings.

To explain these findings, it can be mentioned that the instruments measuring the life quality comprise general and specific questionnaires. General instruments that explore the quality of life can be applicable to unhealthy and healthy individuals. An example for these explanations can be the FS-36 questionnaire which is a widely used instrument (Jacobsen and Jim, 2011). Similarly, the HOQOL-100 questionnaire is another widely used instrument that can be utilized in different cultural contexts as well (Whoqol Group, 1998). 

Application of such instruments can be beneficiary due to possibility of comparing the life quality of healthy and unhealthy individuals. Nonetheless, these instruments fail to assess the impacts of cancer and its therapeutic approaches on the individuals’ signs and symptoms such as nausea, vomiting, pain, and fatigue. This can be perceived as a drawback to the application these instruments. In addition, these instruments may fail to assess other aspects of life quality including cognitive dimension and sexual function that may negatively be influenced as a result of cancer. Therefore, the application of specific instruments can provide the possibility of comprehensive assessment of the life quality and its different aspects. However, these instruments fail to compare the life quality of healthy and unhealthy individuals (Jacobsen and Jim, 2011). To explain more, the selection of appropriate instruments should be based on the study’s purpose. The studies aiming at comparing the life quality of health and unhealthy individuals can use general instruments. Though, the studies with clinical trial design are expected to use specific instruments that evaluate the quality of life (Testa, 1996). 

In the present study, the majority of reviewed articles used specific instruments in order to assess the quality of life while other studies used a combination of both general and specific instruments. This can be perceived as a strength of this study because the combination of different types of the instruments appears to provide more appropriate information (Cornish et al., 2009). 

As to the information concerning the instrument usage, the findings demonstrated that less than half of the studies focused solely on the instrument’s author(s) and the target group. Wheelwright et al., (2013) stated that less than half of the studies out of total 67 reviewed articles explained the reason(s) for the selection of a particular instrument, whereas these information are necessary for every instrument in order to select a proper instrument for research studies. Provision of the reason(s) for choosing an instrument indicates that some particular criteria are taken into account by the researcher(s) prior to the selection of that instrument. Otherwise, an instrument may be chosen that fails to meet the study’s purposes.

In three studied articles, the instruments were translated but no sufficient information regarding the translation process was provided by the researchers (Agha barari et al., 2007; Rad et al., 2016; Bahrami et al., 2013). 

Similarly, a study that investigated the death anxiety and its relation to the life quality of adult females referred merely to the instrument’s validity (Content and construct) and reliability (Test-retest and correlation coefficient) with no detailed explanation on methodology and results (Bahrami et al., 2013). 

In a systematic review, Claassen et al., (2011) surveyed 53 articles which were conducted on the patients with lung cancer, of which 35 articles utilized a translated version of the instrument which was unrepresentative of the population under consideration . However, experts highlight the significance of appropriate translation and psychometrics of the questionnaires in order to gain authentic data which are capable of being compared with other studies (Afrasiabifar et al., 2006). Translation of questionnaire by researchers without taking account of the panel of expert translators, as an example, interfere with access to a standard questionnaire which is an appropriate representative of a society (Rahman et al., 2003). Therefore, it is essential to develop standard questionnaires which are translated from the original language into the target language and vice versa, have undergone psychometric tests and possess final report (Pashandi et al., 2009). 

A review of studies exploring the validity and reliability of instruments indicated that most studies failed to provide information concerning psychometric properties of the instruments. Wheelwright et al., (2013), in their study reviewing 67 articles concerning the life quality of cancer patients with cachexia, found out that all studies were poorly written in this context . Fitzsimmons et al., (2009) also explored the life quality of elderly patients with cancer and stated that only six studies elaborated on the psychometric properties of instruments. Also, little evidences were provided regarding cultural validity of the instruments and most studies solely referred to the instruments’ psychometrics in previous studies . In this regard, a study by Claassen et al., (2011) declared that %34 of the studies provided no explanation concerning the validity and reliability of instruments. On the other hand, the studies studying the cultural validity of instruments were scarce. These are consistent with the findings of the current study and highlight this matter that the researchers appear to underestimate the importance of psychometric properties of the instruments in their studies and that this matter, to some extent, has been disregarded by the authors and reviewees recently. Whereas, the instruments that are used in studies are expected to have optimal psychometric properties (Fallowfield, 1995). In addition, it seems necessary that enough attention be paid to the validity and reliability of the life quality questionnaires. As a results, the queries will cover all aspects of the life quality and will provide steady and actual results under frequent assessments that are indications of the instrument’s optimal strength (Tamburini, 2001).

In conclusion, a variety of instruments have been utilized by the researchers so far that explore the life quality of cancer patients. Some studies utilized a general instrument for this purpose and the majority provided insufficient information concerning the instruments and their psychometrics or used an inappropriate method to assess the instruments’ validity and reliability. Given the importance of psychometric properties of the instruments and its impact on the findings emerging from different studies, it seems necessary that more attention be paid to the validity and reliability of instruments prior to conducting the research studies. Also, it should be emphasized that the application of valid and reliable instruments can increase the strength of studies and their outputs. Therefore, it is recommended that the researchers pay enough attention to the selection of standard instruments and prioritize the instruments’ validity and reliability in their future researches. Given the importance of evaluating the life quality of patients with cancer and according to the findings of the present study, appropriate interventions can be planned and implemented to improve the quality of life of patients in different aspects. Therefore, the selection of a valid and reliable instrument is of pivotal importance. Additionally, it is recommended that the instruments with optimal psychometrics which are compatible with the socio-cultural context of our country be classified according to the type of illness. Publication of these instruments in relevant texts and journals concerning the patients with cancer can provide the researchers and scholars with the opportunity to perform a complete and accurate evaluation and thereby obtain reliable findings. 

## References

[B1] Abedi HA, Alavi M, Musarezaie A, Mazroie-Sebdani A (2014). The effect of logotherapy on cancer patient’s quality of life. J Res Behav Sci.

[B2] Afrasiabifar A, Yaghmaei F, Abdoli S, Abed SZ (2006). Research tool translation and cross-cultural adaptation. FNMQ.

[B3] Agha barari M, Ahmadi F, Mohammadi E, Hagizadeh E, Varvarani A (2007). Physical, emotional and social dimension of quality of life among breast cancer women under chemotherapy. IJNR.

[B4] Ayatollahi H (2013). Quality of life in breast cancer patients: Study in the Omid cancer research center– Urmia. IJBD.

[B5] Bahrami M (2016). Iranian nurses perceptions of cancer patients quality of life. Iran J Cancer Prev.

[B6] Bahrami N, Moradi M, Soleimani M, Kalantari Z, Hosseini F (2013). Death anxiety and its relationship with quality of life in women with Cancer. IJN.

[B7] Baljani E, Kazemi E, Amanpour E, Tizfahm T (2011). Asurvey on relationship between relegion ,spritual wellbeing,hope and quality of life in patients with cancer. EBCJ.

[B8] Bialocerkowski AE, Bragge P (2008). Measurement error and reliability testing: Application to rehabilitation. IJTR.

[B9] Bonomi AE, Patrick DL, Bushnell DM, Martin M (2000). Validation of the United States’ version of the world health organization quality of life (WHOQOL) instrument. J Clin Epidemiol.

[B10] Burckhardt CS, Anderson KL (2003). The quality of life scale (QOLS): reliability, validity, and utilization. Health Qual Life Outcomes.

[B11] Cook DA, Beckman TJ (2006). Current concepts in validity and reliability for psychometric instruments: theory and application. Am J Med.

[B12] Cornish D, Holterhues C, Van de Poll-Franse LV, Coebergh JW, Nijsten T (2009). A systematic review of health-related quality of life in cutaneous melanoma. Ann Oncol.

[B13] Damm K, Roeske N, Jacob C (2013). Health-related quality of life questionnaires in lung cancer trials: a systematic literature review. Health Econ Rev.

[B14] Davoodi A, Rezazadeh H, Somi M (2012). Effect of educating self-care program on quality of life in patients with gastric cancer after gastrectomy in Tabriz Hospitals. Med J Tabriz Univ Med Sci Health Serv.

[B15] DeVon HA, Block ME, MoyleWright P (2007). A psychometric toolbox for testing validity and reliability. J Nurs Scholarsh.

[B16] Drost EA (2011). Validity and reliability in social science research. ERIC.

[B17] Fallowfield L (1995). Quality of quality-of-life data. Lancet.

[B18] Fazel A, Tirgari B, Mokhber N, Koushyar M, Esmaily H (2008). The effect of mastectomy on mood and quality of life in breast cancer patients. JSSU.

[B19] Ferlay J, Soerjomataram I, Dikshit R (2015). Cancer incidence and mortality worldwide: sources, methods and major patterns in GLOBOCAN 2012. Int J Cancer.

[B20] Fitzsimmons D, Gilbert J, Howse F (2009). A systematic review of the use and validation of health-related quality of life instruments in older cancer patients. EJC.

[B21] Ghavam-Nasiri M, Heshmati Nabavi F, Anvari K (2012). The effect of individual and group self-care education on quality of life in patients receiving chemotherapy: A randomized clinical trial. IJME.

[B22] Glaassens L, Van Meerbeeck J, Coens C (2011). Health-related quality of life in non–small-cell lung cancer: An update of a systematic review on methodologic issues in randomized controlled trials. J Clin Oncol.

[B23] Golafshani N (2003). Understanding reliability and validity in qualitative research. TQR.

[B24] Haghighat S (2013). The effect of reflexology on quality of life of breast cancer patients during chemotherapy. IJBD.

[B25] Hasanvand S, Ashktorab T, Jafari Z, Salmani N, Safariyan Z (2015). Cancer-related fatigue and its association with health-related quality of. Adv Nurs Midwife.

[B26] Haynes SN, Richard D, Kubany ES (1995). Content validity in psychological assessment: A functional approach to concepts and methods. Psychol Assess.

[B27] Heravi Karimovi M, Pourdehqan M, Jadid Milani M, Foroutan S, Aieen F (2006). Study of the effects of group counseling on quality of sexual life of patients with breast cancer under chemotherapy at Imam Khomeini Hospital. IMEMR.

[B28] Heydari S, Salahshourian-fard A, Rafii F, Hoseini F (2009). Correlation of perceived social support from different supportive sources and the size of social network with quality of life in cancer patients. IJN.

[B29] Jacobsen PB, Davis K, Cella D (2002). Assessing quality of life in research and clinical practice. Oncology.

[B30] Jacobsen PB, Jim HS (2011). Consideration of quality of life in cancer survivorship research. Cancer Epidemiol Biomarkers Prev.

[B31] Karbaschi K, Zareiyan A, Dadghari F, Siyadati SA (2015). The effect of self-care program based on Orem’s theory on quality of life of cancer patients undergoing chemotherapy in military personnel. Mil Caring Sci.

[B32] Leppert W, Gottwald L, Forycka M (2015). Clinical practice recommendations for quality of life assessment in patients with gynecological cancer. Prz Menopauzalny.

[B33] Mardani Hamule M, Shahraki Vahed A (2010). Relationship between mental health and quality of life in cancer patients. JSSU.

[B34] Mikaili P (2014). Evaluation of the effect of chemotherapy on functional scales of quality of life of patient with breast cancer. IJBD.

[B35] Momeni M, Ghanbari A (2011). Comparison of specific quality of life between urban and rural colorectal cancer patients. Govaresh.

[B36] Monfared A, Pakseresht S, Ghanbari Khanghah A, Atrkar-Roshan Z (2013). Health-related quality of life and its related factors among women with breast cancer. J Holist Nurs.

[B37] Musarezaie A, Ghasemi TM, Esfahani HN (2012). Investigation the quality of life and its relation with clinical and demographic characteristics in women with breast cancer under chemotherapy. Int J Prev Med.

[B38] Pashandi Sh, Khaghanizade M, Ebadi AB (2009). Review of translation and cultural adaptation process of questionnaires. Educ Strategy Med.

[B39] Polit DF, Beck CT (2006). The content validity index: are you sure you know what’s being reported? critique and recommendations. Res Nurs Health.

[B40] Polit DF, Beck CT, Owen SV (2007). Is the CVI an acceptable indicator of content validity? Appraisal and recommendations. Res Nurs Health.

[B41] Poorkiani M, Hazrati M, Abbaszadeh A (2010). Dose a rehabilitation program improve quality of life in breast cancer patients. Payesh.

[B42] Rad M, Borzoee F, Mohebbi M (2016). The effect of Humor therapy on fatigue severity and quality of life in breast cancer patients undergoing external radiation therapy. JZUMS.

[B43] Rahman A, Iqbal Z, Waheed W, Hussain N (2003). Translation and cultural adaptation of health questionnaires. J Pakistan Med Assoc.

[B44] Rezaei R, Saatsaz S, Haji Hosseini F, Sharifnia S, Nazari R (2013). Quality of life in gynecologic cancer patients before and after chemotherapy. JBUMS.

[B45] Rouhollahi MR, Mohagheghi MA, Mohammadrezai N (2014). Situation analysis of the National Comprehensive Cancer Control Program (2013) in the I R of Iran; assessment and recommendations based on the IAEA IMPACT mission. Arch Iran Med.

[B46] Sadat Aghahosseini Sh, Rahmani A, Abdollahzadeh F, Asvadi Kermani I (2012). Life quality of cancer patient with or without self awareness. J Gorgan Univ Med Sci.

[B47] Samiei Siboni F, Moni Anoosheh M, Alhani F (2010). The study of the effects of group counseling on symptom scales of QOL of patients with breast cancer undergoing chemotherapy. IJBD.

[B48] Shahsavari H, Matory P, Zare Z (2015). Correlation between quality of life and individual factors in the patients with breast cancer in Seied Alshohada Hospital in Isfahan in 2013. Community Health J.

[B49] Shariati AA, Salehi M, Ansari M, Latifi SM (2011). Survey the effect of Benson relaxation intervention on quality of life (QOL) in breast cancer patients undergoing chemotherapy. Sci Med J Ahwaz Jundishapur Univ Med Sci.

[B50] Sharif F, Abshorshori N, Tahmasebi S (2010). The effect of peer-led education on the life quality of mastectomy patients referred to breast cancer-clinics in Shiraz, Iran 2009. Health Qual Life Out.

[B51] Shirinabadi Farahani A, Rassouli M, Yaghmaei F, Alavi Majd H (2015). Index for selecting an appropriate instrument to conduct research in health sciences: Introducing the COSMIN checklist. JHPM.

[B52] Tabari F, Zakeri Moghadam M, Bahrani N, Monjamed Z (2007). Evaluation of the quality of life in newly recognized cancer patients. Hayat.

[B53] Tadele N (2015). Evaluation of quality of life of adult cancer patients attending Tikur Anbessa specialized referral hospital, Addis Ababa Ethiopia. Ethiop J Health Sci.

[B54] Taghadosi M, Fahimifar A (2014). Effect of life review therapy with spiritual approach on the life quality among cancer patients. Feyz.

[B55] Tamburini M (2001). Health-related quality of life measures in cancer. Ann Oncol.

[B56] Tavakol M, Dennick R (2011). Making sense of Cronbach’s alpha. Int J Med Educ.

[B57] Testa MA (1996). Simonson DC Assessment of quality-of-life outcomes. N Engl J Med.

[B58] Theofilou P (2013). Quality of life: definition and measurement. EJOP.

[B59] Tork Zahrani S, Rastegari L, Khoda Karami N, Mohebbi P (2012). Relationship between quality of life and social support in women treated for cervical cancer. PCNM.

[B60] Torkzahrani S, Rastegari L, Khodakarami N, Akbarzadeh-Baghian A, Alizadeh K (2013). Quality of life and its related factors among Iranian cervical cancer survivors. Iran Red Crescent Med J.

[B61] Ubel PA, Loewenstein G, Jepson C (2003). Whose quality of life? A commentary exploring discrepancies between health state evaluations of patients and the general public. Qual Life Res.

[B62] Üstündağ S, Zencirci AD (2015). Factors affecting the quality of life of cancer patients undergoing chemotherapy: A questionnaire study. Pac J Oncol Nurs.

[B63] Wells GA, Russell AS, Haraoui B, Bissonnette R, Ware CF (2011). Validity of quality of life measurement tools-from generic to disease-specific. J Rheumatol Suppl.

[B64] Wheelwright S, Darlington AS, Hopkinson JB (2013). A systematic review of health-related quality of life instruments in patients with cancer cachexia. Support Care Cancer.

[B65] Whoqol Group (1998). Development of the world health organization WHOQOL-BREF quality of life assessment. Psychol Med.

[B66] Yaghmaei F (2006). Critical review of psychometric properties in research questionnaires. Fac Nurs Midwif Qurterl.

[B67] Yazdani F (2015). The effects of Yoga on symptom scales quality of life in breast cancer patients undergoing radiotherapy. IJBD.

[B68] Zeighami Mohammadi S, Ghaffari F (2009). Sexual dysfunction and its correlation with quality of life among women affected with cancer. IJOGI.

[B69] Zeighami Mohammedi Sh, Hushmand P, Jafari F, Esmaiely H, Kooshyar MM (2008). The relationship of anemia with severity of fatigue and quality of life in cancer patient undergoing chemotherapy. Sci J Hamadan Nurs Midwifery Fac.

